# Small Airway Dysfunction Assessed by Impulse Oscillometry in Children with Congenital Adrenal Hyperplasia: A Preliminary Case–Control Study

**DOI:** 10.3390/jcm15156029

**Published:** 2026-08-03

**Authors:** Ercan Yılmaz, Erdem Topal, İsmail Dündar, Zeynep Yamancan Yılmaz, Nezihe Koker Ozer, Eda Kaya, Emine Çamtosun

**Affiliations:** 1 Department of Pediatric Allergy and Immunology, Faculty of Medicine, Inonu University, Malatya 44210, Turkey; ercanyilmz44@gmail.com (E.Y.); erdemtopal44@gmail.com (E.T.); nezihe.koker@inonu.edu.tr (N.K.O.); 2 Department of Pediatric Endocrinology, Faculty of Medicine, Inonu University, Malatya 44210, Turkey; zynpymncan@hotmail.com (Z.Y.Y.); eda0688@gmail.com (E.K.);

**Keywords:** congenital adrenal hyperplasia, impulse oscillometry, small airway dysfunction

## Abstract

**Background**: Congenital adrenal hyperplasia (CAH) is associated with chronic hormonal dysregulation and prolonged glucocorticoid exposure, which may contribute to alterations in pulmonary physiology. However, small airway function in children with CAH has not been adequately investigated. This study aims to evaluate small airway function in children with congenital adrenal hyperplasia (CAH) using impulse oscillometry system (IOS) and to compare the findings with those of healthy controls. **Methods**: This cross-sectional case–control study included 27 children with CAH and 27 age- and sex-matched healthy controls. IOS measurements, including resistance at 5 Hz (R5), resistance at 20 Hz (R20), peripheral airway resistance (R5–R20), reactance at 5 Hz (X5) and 20 Hz (X20), reactance area (AX), and resonance frequency (Fres), were obtained in all participants. Demographic, clinical, laboratory, and treatment-related characteristics were also recorded. **Results**: The median age of the CAH group was 14 years, and 66.7% were female. Compared with controls, children with CAH demonstrated significantly higher R5 values [0.55 vs. 0.38 kPa·s/L, *p* = 0.031], increased R5–R20 values [0.21 vs. 0.12 kPa·s/L, *p* = 0.009], and elevated AX values [2.05 vs. 1.02 kPa·s/L, *p* = 0.001]. In addition, X5 values were significantly more negative in the CAH group [−0.16 vs. −0.10 kPa·s/L, *p* = 0.001]. No significant differences were observed in R20, X20, and Fres parameters. Based on the z-scores calculated using the reference equations implemented in the device software, abnormal oscillometric findings were most frequently observed for X5 (29.6%), followed by R5–R20 (25.9%) and R5 (22.2%). **Conclusions**: Children with CAH exhibited IOS abnormalities suggestive of subclinical small airway dysfunction despite the absence of overt respiratory disease. IOS may serve as a sensitive tool for the early detection of small airway involvement in pediatric CAH. Further prospective studies with larger sample sizes are warranted.

## 1. Introduction

Congenital Adrenal Hyperplasia (CAH) is a heterogeneous group of disorders with autosomal recessive inheritance, resulting from enzyme deficiencies in adrenal steroid synthesis. The condition is primarily characterized by impaired cortisol synthesis and may lead to varying degrees of aldosterone deficiency and androgen excess. The global prevalence of classic CAH is reported to be approximately 1 in 10,000–15,000 live births [1]. The most common form is 21-hydroxylase deficiency, caused by mutations in the CYP21A2 gene, accounting for approximately 90–95% of cases [2]. Less commonly, deficiencies in 11β-hydroxylase, 17α-hydroxylase and other steroidogenesis enzymes may be observed.

Congenital adrenal hyperplasia is not merely a disease affecting the endocrine system; it can lead to multisystemic consequences due to chronic hormonal imbalance, long-term glucocorticoid therapy and metabolic effects [3]. In recent years, the effects of endocrine disorders on pulmonary physiology have attracted increasing attention. In particular, it is thought that chronic inflammation, hormonal imbalance and metabolic changes may affect airway function [4].

Glucocorticoids play a fundamental role in fetal lung development, surfactant synthesis, the maintenance of epithelial integrity, and the regulation of airway inflammation [5]. Consequently, both cortisol deficiency and long-term exogenous glucocorticoid use can affect airway physiology. Furthermore, it has been suggested that chronic androgen excess may also influence bronchial smooth muscle tone and inflammatory responses [6].

Small airway dysfunction in childhood has become a significant area of research in recent years. Early functional changes in the small airways often cannot be detected by conventional spirometry and are referred to as the ‘silent zone’ [7]. Consequently, there is a need for methods capable of assessing distal airway function more sensitively.

The impulse oscillometry system (IOS) is a non-invasive method that assesses airway resistance and reactance during tidal breathing. IOS has gained importance particularly in pediatric patients due to its ease of application, the reduced need for patient cooperation, and its ability to accurately assess small airway function [8]. Among the IOS parameters, R5 indicates total airway resistance, R20 indicates central airway resistance, and R5–R20 indicates peripheral airway resistance. The X5 and AX parameters, meanwhile, provide information on distal airway elastic properties and reactance abnormalities [9].

Small airway dysfunction has been demonstrated using IOS in various pediatric conditions such as asthma, obesity-related airway diseases, bronchiolitis obliterans and cystic fibrosis [10,11,12]. However, to the best of our knowledge, there are no studies evaluating airway function using IOS in children with CAH. Most existing studies rely on conventional spirometry, and peripheral airway function has not been sufficiently investigated. It is thought that chronic hormonal imbalance, inflammation, and long-term steroid treatment in CAH may cause subclinical changes in the small airways. Therefore, the identification of patients with early peripheral airway involvement may be clinically significant.

The aim of this study was to evaluate small airway function using IOS in children diagnosed with CAH and to compare the results with a healthy control group.

## 2. Materials and Methods

### 2.1. Study Design and Patient Population

This cross-sectional case–control study was conducted in the pediatric endocrinology and pediatric allergy-immunology clinics of a tertiary university hospital. The study included a total of 27 pediatric patients aged 3–18 years who were being followed up with a diagnosis of congenital adrenal hyperplasia, along with 27 healthy controls matched for age and sex. Immediately after obtaining ethics committee approval, potentially eligible patients were identified from the pediatric endocrinology outpatient clinic database and contacted by telephone. Families willing to participate were invited to the clinic for evaluation. During the study visit, demographic and clinical data were recorded using standardized case report forms, and all respiratory assessments were completed on the same day under the supervision of the study investigators.

The congenital adrenal hyperplasia group comprised patients with 21-hydroxylase deficiency, 11β-hydroxylase deficiency, 17α-hydroxylase deficiency, and other rare enzyme defects. Care was taken to ensure that patients were in a clinically stable condition, and individuals experiencing an acute adrenal crisis were excluded from the assessment. Furthermore, children with a history of chronic lung disease, congenital heart disease, neuromuscular disorders, prematurity, or respiratory tract infection within the preceding four weeks were excluded. The control group consisted of healthy children attending pediatric outpatient clinics who had no known chronic systemic or pulmonary disease. Care was taken to ensure that the control group had no history of chronic medication use, previous chronic lung disease, congenital heart disease, or active infection.

### 2.2. Clinical Assessment

Detailed demographic and clinical characteristics of all participants were recorded. Age, gender, exposure to smoking, history of consanguineous marriage, history of adrenal crisis, concomitant atopic diseases, family history of atopic diseases, current treatment regimen, and clinical status were assessed. Patients’ height and weight measurements were taken using standard methods, and height z-scores and weight z-scores were calculated according to age and gender. Anthropometric assessments were performed in accordance with national reference data [13] In addition, patients’ current clinical status was assessed as either stable or uncontrolled disease. A history of adrenal crisis and disease complications was investigated.

### 2.3. Assessment of Laboratory and Treatment Characteristics

Laboratory data for all patients were obtained retrospectively from patient records. Eosinophil counts were recorded from peripheral complete blood counts, and total serum immunoglobulin E (IgE) levels were assessed based on the available data. The treatment agents used by patients with CAH were examined in detail. Whilst all patients were receiving hydrocortisone treatment, patients also receiving fludrocortisone, antihypertensive treatment, antiandrogen treatment, oral contraceptive/estrogen treatment, aromatase inhibitor and testosterone treatment were recorded. The daily hydrocortisone dose was calculated in mg/m^2^/day, and patients’ current treatment regimens were assessed. Furthermore, treatment characteristics were analyzed in detail within the scope of the study, taking into account the potential pulmonary effects of long-term steroid use.

### 2.4. Assessment of Airway Functions Using Impulse Oscillometry System

Airway resistance and reactance were assessed using an impulse oscillometry system (IOS) (Jaeger MasterScreen IOS, CareFusion, Yorba Linda, CA, USA). The device was calibrated daily prior to testing using a standard 3-L syringe and reference resistance, in accordance with the manufacturer’s instructions. All IOS parameters were expressed as absolute values and, where available, as percent-predicted values generated by the Jaeger MasterScreen IOS system using SentrySuite software, version 3.20 (Vyaire Medical GmbH, Höchberg, Germany) using age-, sex-, height-, and ethnicity-specific reference equations. 

All measurements were performed in accordance with the technical standards for respiratory oscillometry published by the European Respiratory Society (ERS) and supported by the American Thoracic Society [14]. Participants were instructed to avoid heavy meals, vigorous exercise, and short-acting bronchodilators for at least 4–6 h prior to testing. Measurements were conducted in a quiet environment with participants in a seated position, wearing a nose clip and maintaining a neutral head position. To minimize upper airway artifact, participants firmly supported their cheeks and the floor of the mouth with their hands.

During the procedure, subjects were asked to breathe normally (tidal breathing) through a mouthpiece connected to the IOS device for approximately 30–45 s, ensuring a tight seal around the mouthpiece. At least three technically acceptable measurements were obtained from each participant. Measurement reproducibility was assessed using the coefficient of variation (CV) of R5, and only measurements with an R5 CV < 15% were accepted for analysis. Measurements were considered acceptable if they met the following criteria: absence of artifacts such as coughing, swallowing, vocalization, air leakage, or irregular breathing; a stable tidal breathing pattern; and coherence values of ≥0.80 at 5 Hz and ≥0.90 at frequencies ≥ 10 Hz. The mean values of the acceptable measurements were used for analysis.

The IOS parameters evaluated included total airway resistance at 5 Hz (R5), central airway resistance at 20 Hz (R20), and the difference between R5 and R20 (R5–R20), which reflects peripheral (small airway) resistance. Reactance parameters included reactance at 5 Hz (X5), reactance area (AX), and resonance frequency (Fres). Negative X5 values indicate reduced elastic recoil and peripheral airway involvement. In addition to the absolute oscillometric values, z-scores were calculated using the reference equations implemented in the device software, taking into account age, sex, height, and ethnicity where applicable. Abnormal values were defined according to the upper limit of normal (ULN) or lower limit of normal (LLN). For resistance and impedance-derived parameters (R5, R20, R5–R20, AX, and Fres), values with z-scores > +1.645 were considered abnormal, whereas for reactance parameters (X5 and X20), values with z-scores < −1.645 were considered abnormal. The frequency of abnormal oscillometric findings was subsequently determined for each parameter.

### 2.5. Statistical Analysis

Statistical analysis was performed using SPSS Statistics (v.21.0; IBM Corp., Armonk, NY, USA). Normality was assessed using the Kolmogorov–Smirnov test. Descriptive statistics were expressed as frequency and percentage for categorical variables whereas the median (IQR or min-max) was used for quantitative data. Categorical variables were compared using the Pearson’s chi-square test or Fisher’s exact test, and quantitative variables were compared using the Mann–Whitney U test. Two-tailed *p*-values < 0.05 were considered statistically significant. Due to the exploratory design of the study and the limited availability of eligible patients with congenital adrenal hyperplasia, no a priori sample size calculation was performed.

The authors declare that no artificial intelligence (AI) or AI-assisted technologies were used in the conduct of this research or in the preparation of this manuscript.

## 3. Results

### 3.1. Demographic and Clinical Characteristics

A total of 27 children diagnosed with CAH were included in the study. Eighteen of the patients were girls (66.7%) and nine were boys (33.3%). The median age of the participants was 14 years (3–18 years). The median height z-score was −0.38 (−4.1–2.49), the median weight z-score was 0.12 (−3.7–4.5), and the median body mass index z-score was 0.64 (−3.55–3.73). When enzyme deficiency types were assessed, 12 patients (44.4%) had 21-hydroxylase deficiency, 9 patients (33.3%) had 11β-hydroxylase deficiency, 5 patients (18.5%) had 17α-hydroxylase deficiency, and 1 patient (3.7%) had 20–22-desmolase deficiency. In terms of clinical status, 26 patients (96.3%) were stable, whilst 1 patient (3.7%) had uncontrolled disease. No complications were observed in any of the patients. A history of adrenal crisis was present in 2 patients (7.4%), whilst a history of consanguineous marriage was present in 16 patients (59.3%). Exposure to tobacco was observed in 6 patients (22.2%) (Table 1).

### 3.2. Atopy, Laboratory Findings and Treatment Characteristics

Upon assessment of the patients’ atopic characteristics, it was found that 4 patients (14.8%) had a history of asthma, 3 patients (11.1%) had a history of allergic rhinitis, 5 patients (18.5%) had a history of eczema, and 6 patients (22.2%) had a history of urticaria. No history of food or drug allergy was identified in any of the patients. Upon examination of the family history of atopic diseases, it was observed that 3 patients (11.1%) had a family history of asthma, 5 patients (18.5%) had a history of allergic rhinitis, 4 patients (14.8%) had a history of eczema, and 3 patients (11.1%) had a history of drug allergy. Laboratory findings revealed a median eosinophil count of 1.20/mm^3^ (0.1–5.5/mm^3^) and a median total IgE level of 88 IU/mL (18–332 IU/mL). When treatment characteristics were assessed, it was observed that all 27 patients (100%) were using hydrocortisone. In addition, 9 patients (33.3%) were receiving fludrocortisone, 7 patients (25.9%) antihypertensive treatment, 6 patients (22.2%) antiandrogen treatment, 5 patients (18.5%) oral contraceptive/estrogen treatment, 2 patients (7.4%) were receiving an aromatase inhibitor, and 2 patients (7.4%) were receiving testosterone therapy. The median hydrocortisone dose was found to be 12 mg/m^2^/day (range 8–20 mg/m^2^/day) (Table 1).

### 3.3. Impulse Oscillometry System Findings

When the CAH group was compared with the healthy control group, there were 18 female patients (66.7%) in the CAH group and 16 female individuals (59.3%) in the control group; no significant difference was observed in terms of gender distribution (*p* = 0.778). The median age was 14 years (3–18 years) in the CAH group and 13 years (10–16 years) in the control group; no significant difference was observed in terms of age either (*p* = 0.578). Exposure to smoking was present in 6 individuals (22.2%) in the CAH group and in 5 individuals (18.5%) in the control group; no significant difference was found between the two groups (*p* = 1.0). A history of asthma was identified in 4 patients (14.8%) in the CAH group and in 2 individuals (7.4%) in the control group; this difference was not statistically significant (*p* = 0.669).

When impulse oscillometry system parameters were evaluated, the R5 value was found to be significantly higher in the CAH group compared to the control group [0.55 (0.31) kPa·s/L vs. 0.38 (0.17) kPa·s/L, *p* = 0.031]. The R5–R20 value, indicative of small airway resistance, was also significantly higher in the CAH group [0.21 (0.16) kPa·s/L vs. 0.12 (0.05) kPa·s/L, *p* = 0.009]. The AX value was found to be higher in the CAH group compared to the control group [2.05 (1.82) kPa·s/L vs. 1.02 (0.65) kPa·s/L, *p* = 0.001]. Furthermore, the X5 value was found to be more negative in the CAH group [−0.16 (0.09) kPa·s/L vs. −0.10 (0.06) kPa·s/L, *p* = 0.001] (Table 2). However, no significant differences were observed between the groups in terms of the R20, X20 and Fres parameters. R20 values were 0.30 (0.12) kPa·s/L in the CAH group and 0.25 (0.15) kPa·s/L in the control group (*p* = 0.257). X20 values were found to be −0.02 (0.09) kPa·s/L and 0.0 (0.07) kPa·s/L, respectively (*p* = 0.152). Fres values were 21.5 (7.83) Hz in the CAH group and 20.3 (8.64) Hz in the control group, with no statistically significant difference detected (*p* = 0.598) (Table 2).

Based on the z-scores calculated using the reference equations implemented in the device software, abnormal oscillometric findings were most frequently observed for X5 (29.6%), followed by R5–R20 (25.9%) and R5 (22.2%) (Table 3).

## 4. Discussion

In this study, significant IOS abnormalities suggestive of peripheral airway dysfunction were identified in children with congenital adrenal hyperplasia (CAH) compared with healthy controls. In particular, significantly higher R5, R5–R20, and AX values, together with more negative X5 values, support the presence of small airway dysfunction. These findings were further reinforced by the z-score analysis, which demonstrated that a substantial proportion of children with CAH exhibited abnormal oscillometric values exceeding the GLI-defined limits of normality, particularly for X5, R5–R20, and R5. The concordance between conventional IOS parameters and GLI-based z-score abnormalities strengthens the evidence that these findings reflect true peripheral airway dysfunction rather than normal physiological variation.

To our knowledge, this study is one of the first to evaluate small airway function in pediatric patients with CAH using the impulse oscillometry system (IOS). The number of studies examining the relationship between CAH and pulmonary function in the literature is quite limited, and the majority of existing studies have focused on classical spirometric parameters. However, spirometry primarily assesses large airways and may be inadequate for detecting early functional changes in small airways [7,15]. Particularly in pediatric populations, peripheral airway dysfunction may remain clinically silent for prolonged periods and may only be detected using sensitive techniques such as impulse oscillometry before spirometric abnormalities become apparent [8].

Impulse oscillometry system offers significant advantages in the pediatric age group as it can assess airway resistance and reactance during tidal breathing. Unlike spirometry, it does not require a forced expiratory maneuver, which enhances its applicability in young children [8]. Furthermore, IOS is recognized as a valuable method, particularly for the early detection of distal airway obstruction, as it can assess central and peripheral airways separately [16,17,18].

In our study, the significantly elevated R5–R20 values are particularly noteworthy. The R5–R20 parameter reflects the difference between total airway resistance at low frequencies and central airway resistance at high frequencies, and is considered one of the most sensitive indicators of peripheral airway resistance. As heterogeneous narrowing of the small airways and peripheral obstruction increase, R5–R20 values rise [19].

In previous studies, elevated R5–R20 values have been associated in particular with asthma, obesity-related airway disease, bronchiolitis obliterans and early-onset inflammatory airway diseases [4,8,9]. Shi et al. demonstrated that elevated R5–R20 values in childhood asthma are associated with poor asthma control and peripheral airway inflammation [9]. Similarly, Galant et al. reported that IOS parameters may be more sensitive than spirometry, particularly in detecting mild airway obstruction [20]. In our study, the high R5–R20 values detected in the CAH group suggest the presence of subclinical peripheral airway involvement in these patients. Interestingly, these findings were observed in children without clinically apparent respiratory disease. Furthermore, the absence of significant differences between groups regarding asthma history and exposure to tobacco smoke suggests that IOS abnormalities cannot be explained solely by coexisting atopic conditions.

In our study, not only the R5–R20 values but also the finding of high AX values and more negative X5 values supports peripheral airway involvement. AX is a sensitive parameter reflecting reactance abnormalities and elastic load in the distal airways [16]. In particular, high AX values are associated with early small airway involvement in inflammatory airway diseases [21]. X5, on the other hand, reflects the elastic properties of the lungs and distal airways. More negative X5 values are associated with increased airway stiffness and impaired elasticity in the distal airways [9]. This suggests that subclinical airway remodeling and early-stage small airway dysfunction may be present in COPD.

The underlying mechanisms of small airway dysfunction in CAH are not fully understood. However, it is thought that chronic hormonal imbalance, prolonged glucocorticoid exposure and systemic inflammation may play a role. Glucocorticoids play a fundamental role in lung development, surfactant synthesis and the suppression of airway inflammation [5]. Physiological cortisol deficiency or long-term supraphysiological steroid therapy may affect airway elasticity and inflammatory responses. Furthermore, it has been reported that the chronic androgen excess in CAH may also affect bronchial smooth muscle tone and airway reactivity [6]. Studies have shown that sex steroids can modulate airway inflammation and bronchial hyperreactivity [6,22]. Therefore, it is conceivable that the hormonal environment in CAH may lead to structural or functional changes in the small airways.

The fact that no significant differences were observed in IOS parameters reflecting central airways, such as R20 and X20, in our study suggests that airway obstruction develops predominantly in the peripheral airways. Whilst the R20 parameter primarily indicates resistance in the large and central airways, X20 reflects the reactance characteristics of the central airways [16]. The absence of significant changes in these parameters suggests that the large airways are relatively preserved and that functional changes in the early stages are concentrated in the distal airways. As the diameters of small airways are less than 2 mm, their total contribution to airflow is limited, and they can therefore remain clinically silent for a long time [7]. This situation has been defined in the literature as the ‘silent zone of the lung’. Inflammation, early remodeling or loss of elasticity in the peripheral airways may not initially be detected by conventional spirometry or large airway parameters [15]. Consequently, methods with high peripheral airway sensitivity, such as IOS, offer significant advantages in demonstrating early changes.

An interesting finding was that R5–R20, X5, and AX differed between groups, whereas Fres did not. This pattern may reflect the different physiological properties of these IOS indices. R5–R20, X5, and AX are generally considered more sensitive to early peripheral airway dysfunction, while Fres tends to increase only when abnormalities in respiratory system mechanics become more pronounced. Therefore, the lack of a significant difference in Fres does not necessarily contradict the presence of subtle small airway abnormalities but may instead suggest that these changes were relatively mild in the present cohort.

The z-score analysis further supports the presence of subtle peripheral airway abnormalities in children with congenital adrenal hyperplasia. Nearly one-third of patients demonstrated an abnormally reduced X5, while approximately one-quarter had abnormal R5–R20 and R5 values according to GLI reference equations [14,23]. In contrast, abnormalities in central airway indices such as R20 and X20 were relatively uncommon. This pattern is consistent with preferential involvement of the peripheral rather than the central airways and is in agreement with the group comparisons based on continuous IOS measurements. Although only a subset of patients exceeded the conventional upper or lower limits of normal, the z-score analysis provides clinically meaningful information by identifying individuals with objectively abnormal airway mechanics rather than merely demonstrating shifts in group mean values. These findings indicate that clinically relevant small airway dysfunction may already be present in a proportion of children with congenital adrenal hyperplasia despite the absence of overt respiratory disease.

Although no significant differences were observed between groups regarding asthma history or environmental tobacco smoke exposure, the relatively small sample size limited the statistical power to evaluate the potential influence of these and other coexisting atopic conditions, including allergic rhinitis and eczema, on IOS parameters. Therefore, the possible confounding effects of these factors cannot be excluded, and the findings should be interpreted with caution and confirmed in larger studies with adequate power to adjust for potential confounders.

The strengths of our study include the detailed assessment of small airway function using impulse oscillometry, the inclusion of an age- and sex-matched control group, and the comprehensive characterization of the clinical features of children with congenital adrenal hyperplasia. Nevertheless, several limitations should be acknowledged. First, the relatively small sample size and the cross-sectional design limited statistical power and precluded causal inference. Second, spirometric measurements, hormonal control parameters, and inflammatory biomarkers were not available for all participants, preventing a comprehensive evaluation of pulmonary function and limiting assessment of the potential influence of disease activity and hormonal control on IOS findings. Third, adjustment for potential confounding variables, including coexisting atopic conditions and environmental tobacco smoke exposure, was not performed because of the limited sample size; therefore, their contribution to the observed IOS abnormalities cannot be excluded. Accordingly, our findings should be considered preliminary and hypothesis-generating and require confirmation in larger prospective studies incorporating comprehensive pulmonary function testing, biochemical assessment, and multivariable analyses.

## 5. Conclusions

In this study, a significant deterioration in peripheral airway parameters assessed by IOS was observed in children with CAH. Increased R5, R5–R20 and AX values support the presence of small airway dysfunction. These findings suggest that CAH may cause subclinical pulmonary effects in the absence of clinically apparent respiratory disease. This association requires validation in prospective studies with larger sample sizes.

## Figures and Tables

**Table 1:** Demographic, atopic, and treatment characteristics of children diagnosed with congenital adrenal hyperplasia (n:27).

Variables	n (%)
Sex, female	18 (66.7)
Age, median (min–max), years	14 (3–18)
Height z score, median (min–max)	−0.38 (−4.1–2.49)
Weight z score, median (min–max)	0.12 (−3.7–4.5)
Body mass index z score, median (min–max)	0.64 (−3.55–3.73)
Enzyme deficiency type	
21-hydroxylase	12 (44.4)
11β-hydroxylase	9 (33.3)
17α-hydroxylase	5 (18.5)
20–22 Desmolase	1 (3.7)
Current clinical status	
Stable	26 (96.3)
Complications developed	-
Uncontrolled	1 (3.7)
History of adrenal crisis	2 (7.4)
Consanguineous marriage	16 (59.3)
Smoking exposure	6 (22.2)
Presence of atopy in the patient	
Asthma	4 (14.8)
Atopic rhinitis	3 (11.1)
Eczema history	5 (18.5)
Food allergy history	-
Drug allergy history	-
Urticaria	6 (22.2)
Family history of atopy	
Asthma	3 (11.1)
Rhinitis	5 (18.5)
Eczema history	4 (14.8)
Drug allergy history	3 (11.1)
Laboratory findings	
Eosinophil count/mm^3^, median (min–max)	1.20 (0.1–5.5)
Total Ig E, median (min–max), IU/mL	88 (18–332)
Treatment	
Hydrocortisone	27 (100)
Fludrocortisone	9 (33.3)
Antihypertensive	7 (25.9)
Anti-androgen	6 (22.2)
Oral contraceptives/estrogen	5 (18.5)
Aromatase inhibitor	2 (7.4)
Testosterone	2 (7.4)
Hydrocortisone dosage (mg/m^2^/day)	12 (8–20)

**Table 2:** Comparison of Demographic Characteristics and Distribution of Oscillometric Measurements in the Congenital Adrenal Hyperplasia and Healthy Control Groups.

Variables	Congenital Adrenal Hyperplasia Group (n:27)	Healthy Control Group (n:27)	*p*-Value
Gender, female, n (%)	18 (66.7)	16 (59.3)	0.778
Age, median (min–max), years	14 (3–18)	13 (10–16)	0.578
Exposure to smoking, n (%)	6 (22.2)	5 (18.5)	1.0
Asthma, n (%)	4 (14.8)	2 (7.4)	0.669
R5 (kPa·s/L), Interquartile range	0.55 (0.31)	0.38 (0.17)	0.031
R20 (kPa·s/L), Interquartile range	0.30 (0.12)	0.25 (0.15)	0.257
R5–R20 (kPa·s/L), Interquartile range	0.21 (0.16)	0.12 (0.05)	0.009
X5 (kPa·s/L), Interquartile range	−0.16 (0.09)	−0.1 (0.06)	0.001
X20 (kPa·s/L), Interquartile range	−0.02 (0.09)	0.0 (0.07)	0.152
AX (kPa/L), Interquartile range	2.05 (1.82)	1.02 (0.65)	0.001
Fres (Hz), Interquartile range	21.5 (7.83)	20.3 (8.64)	0.598

**Table 3:** Prevalence of Abnormal Impulse Oscillometry Parameters Based on z-Score Thresholds in Children with Congenital Adrenal Hyperplasia (n:27).

Parameters	ULN/LLN Criterion	Frequency of Abnormality n (%)
R5	z > +1.645	6 (22.2)
R20	z > +1.645	3 (11.1)
R5–R20	z > +1.645	7 (25.9)
AX	z > +1.645	5 (18.5)
Fres	z > +1.645	1 (3.7)
X5	z < –1.645	8 (29.6)
X20	z < –1.645	2 (7.4)

ULN: Upper limit of normal; LLN: Lower limit of normal. Abnormal values were defined as z-scores > +1.645 for R5, R20, R5–R20, AX, and Fres, and z-scores < −1.645 for X5 and X20.

## Data Availability

The data presented in this study are available on request from the corresponding author.
